# Three weeks of mental training changes physiological outcomes during a time trial to exhaustion

**DOI:** 10.1007/s00421-023-05206-3

**Published:** 2023-05-01

**Authors:** Timothy A. VanHaitsma, Stephen P. Gonzalez, Sten Kajitani, Emma Gabriano, Gavin E. Hoiosen, Michael C. Oldach, Karly L. Kingsley

**Affiliations:** 1 Department of Kinesiology, Westmont College, Santa Barbara, CA USA; 2 Department of Kinesiology, Sport Studies, and Physical Education, Dartmouth College, Hanover, NH USA

**Keywords:** Mental training, Behavioral strategies, Performance, Electromyography, Oxygen consumption, Ventilation

## Abstract

Mental training (MT) can increase endurance performance. The purpose of this study was to examine the minimum dose of mental training needed to increase performance and elucidate the physiological mechanisms underlying this improvement. In a randomized between groups pre-test–post-test design, 33 participants visited the lab on 6 separate days. A VO_2peak_ with ventilatory threshold (VT) was performed on day 1. The subsequent visits consisted of time trials to exhaustion (TTE) performed at 10% above VT. Between visit 3 and 6, the MT group (*n* = 16) watched a video for 10–15 min each day for 3 weeks, while the control group (CON; *n* = 17) did no mental training. Heart rate (HR), rate of perceived exertion (RPE), VAS scores for pain and fatigue, electromyography, and metabolic and neuromuscular data were collected and recorded during the time trials. The GRIT-S and CD-RISC 10 surveys were completed before study days 3 and 6. TTE increased significantly for MT beginning after 2 weeks (10.0 ± 13.1%) with no further change after 3 weeks (10.4 ± 13.2). TTE also significantly decreased during the last TTE for CON (−10.3 ± 12.7). VO_2_, ventilation, and frequency of breathing were significantly reduced in the latter stages of the TTE for MT. EMG was also significantly decreased for MT as compared for CON throughout the trial. Three weeks of mental training improves performance by reducing EMG, decreasing activation of the muscle and reducing metabolic factors during the latter stages of exercise.

## Introduction

Endurance performance relies heavily on the ability to sustain aerobic exercise over prolonged periods of time up to the point of exhaustion, which is traditionally thought to represent the culmination of progressive muscle fatigue (Allen et al. 2008; Amann and Dempsey 2008; Joyner and Coyle 2008; Amann and Secher 2010). In addition to physiological determinants, perceptual and motivational factors affect the endpoint of endurance performance. The psychobiological model suggests that exhaustion occurs when an individual is no longer willing to exert the effort required by a task (Smirmaul et al. 2013; Pageaux 2014). Psychological, or mental, training has been used to improve endurance performance whether using psychological skills training (Birrer and Morgan 2010; McCormick et al. 2015; Van Haitsma et al. 2019), resilience training (Kee and John Wang 2008; Bernier et al. 2009), motivational arousal training (Brehm and Self 1989), self-talk training (Theodorakis et al. 2000; Blanchfield et al. 2014), or mental toughness (Giles et al. 2017) and grit training (Silvia et al. 2013) perhaps increasing the ability to exert effort while on a task. Though performance is increased, the physiological changes that occur with, or due, to psychological, or mental, training and accompany the improvements in performance are currently unknown.

One of the key psychological aspects that supports high performance is the concept of mental toughness. Mental toughness has been defined as having a single-minded focus on excellence even as the demands experienced during competition change (Cowden 2017). A second definition of mental toughness, as determined by interviews with elite athletes, focuses on staying more determined, focused, confident, and in control while undergoing a stressful situation (Jones 2002). From these definitions, there seem to be two sub-categories of mental toughness that can be potentially measured: grit and resilience. Grit, as defined by (Duckworth et al. 2007), focuses on the passion and perseverance one has in the pursuit of a long-term goal. Resilience, in contrast, can be defined in the context of performance as the use of mental processes to cope with potential stressors—whether positive or negative (Fletcher and Sarkar 2012; Galli and Gonzalez 2015). Previously, individuals higher in grit demonstrated a higher coactivation of parasympathetic and sympathetic systems (Silvia et al. 2013), which may lead to an increased cardiac output by slowing heart rate during exercise and thus increasing ventricular filling time and stroke volume (Koizumi et al. 1982).

The duration of mental training needed to elucidate the maximal performance response is also unknown. Recent successful mental training research ranged from a professional soccer paradigm consisting of an initial 1-h imagery session followed by a booster session 6 weeks later increasing grit (Rhodes et al. 2018). Within laboratory settings, psychological interventions have ranged from a 4-day psychological skills training package (Barwood et al. 2007) to an initial 30 min introduction to self-talk followed by 2 weeks of practicing motivational self-talk statements during customary aerobic exercise sessions (Blanchfield et al. 2014). However, there is some evidence that, as length of the mental practice intervention increases, the overall effectiveness of the intervention on performance decreases (Driskell et al. 1994). Likewise, the longer the delay following mental training, the lower the retention of performance benefits (Driskell et al. 1994).

The purpose of this study was to examine the effect of mental training on endurance performance and to determine the minimal dose of mental training needed to bring about an increase in endurance performance while also attempting to determine the physiological changes that would accompany this increased performance. This was done by utilizing a time trial to exhaustion in a controlled laboratory study. Participants performed four time trials, each separated by 1 week, with mental training taking place every day during each week. It was hypothesized, given previous research, that mental training would improve performance, even with as little as 1 week of mental training, and would occur with reciprocal changes in physiological variables such as rectus femoris electromyography, peripheral fatigue as measured through potentiated twitch (*Q*_tw.pot_), or metabolic data.

## Methods

### Participant characteristics

All experimental procedures in this investigation were reviewed and approved by the Westmont College Institutional Review Board (IRB) prior to the beginning of this study and conformed to the principles of the Declaration of Helsinki. The protocols and procedures were explained, and all participants provided written informed consent prior to testing. Participants (*N* = 33) consisted of recreationally trained individuals recruited by word of mouth (males = 21, females = 12; mean ± SD; age 20.7 ± 1.3 years, body weight 72.3 ± 9.3 kg, height 176.5 ± 8.9 cm, peak oxygen consumption (VO_2peak_) 47.9 ± 9.3 ml/kg/min). Six additional participants began the study, but withdrew before completion and were excluded from the analysis. Participants were excluded if any of the following applied: current acute musculoskeletal injury; medications known to interfere with the sympathetic nervous system; unwillingness to comply with training interruptions mandated by the protocol; any uncontrolled chronic health conditions.

### Protocol overview and exercise protocols

The study consisted of a controlled pre-test–post-test design in which participants visited the laboratory on six separate occasions and were randomized into two independent groups using a random number generator (Mental training (MT) *n* = 16; 9 males, control (CON) *n* = 17; 12 males) during the first visit. The first TTE (performed on study day 3) was classified as the pre-training TTE and was compared to the final TTE, classified as post-training, (performed on study day 6) for all data. All exercise tests were conducted in the same location on the same electromagnetically braked cycle ergometer (Excalibur Sport, Lode, Groningen, the Netherlands), with the saddle height adjusted to suit the preference of each participant and maintained for each visit. Each time trial was performed at the same time of day to avoid circadian rhythm differences between trials. Participants were asked to avoid caffeine before each exercise trial and all participants refrained from exercise for 24 h before each trial. None of the participants were trained cyclists to account for any variance cycling experience would add to time to exhaustion nor were there any monetary incentives for participating in or completing the study.

### Graded exercise test protocol

During visit one, each participant completed an incremental cycling test beginning at 100 W (50 W for females) with resistance increasing 5 W every 15 s until volitional exhaustion to establish VO_2peak_ and to determine ventilatory threshold. Participants pedaled at their preferred pedaling rate, and the test was terminated when the cadence dropped below 70 rpm for more than 5 s, despite strong verbal encouragement. Metabolic data were collected using open circuit calorimetry (Vista MX, Vacumed, Ventura, CA). Peak oxygen consumption (VO_2peak_) was recorded as the highest VO_2_ recorded in a 15 s period. Subsequently, the ventilatory equivalent method, or power output corresponding to a systematic increase in the ventilatory equivalent of oxygen (VE/VO_2_) without a concomitant increase in the ventilatory equivalent of carbon dioxide (VE/VCO_2_), was used to determine the power output at ventilatory threshold (Wasserman and McIlroy 1964).

### Time to exhaustion (TTE) protocol

The subsequent five visits consisted of a time to exhaustion test at a wattage of 10% above the determined ventilatory threshold. The time to exhaustion test began with a 5-min warm-up at 100 W (75 W for women). Following the warm-up, participants were asked to cease pedaling while the power was set on the ergometer. Two minutes after the warm-up ended, participants were asked to begin the TTE. They were encouraged to stand for the first 3–5 s of the TTE to start the flywheel spinning, but they were required to stay seated for the remainder of the trial. Time to exhaustion was defined as the time from the onset of pedaling until the point at which cadence had fallen below 70 rpm for more than 5 s. If cadence was below 70 rpm, researchers tapped on the cycle ergometer’s tachometer to alert the participant to increase cadence. No verbal encouragement was provided at any point during the time to exhaustion test to eliminate any external motivation. Heart rate was recorded every minute throughout the time to exhaustion test using a wireless chest strap (Polar Electro Inc., Bethpage, New York, USA).

Visit two was a familiarization session and was separated from visit three by 1 week. Visits three through six were each separated by 7 days during which the MT interventions took place. MT interventions consisted of watching an initial training video immediately following the exercise test during visits 3–5. During the subsequent weeks, MT participants were asked to watch one of three videos each day for the following week (ending with watching all three videos twice during the week, for a total of watching each video six times each over the course of 3 weeks). Prior to visit 3 and visit 6, participants in each group were instructed to complete the CD-RISC 10 and GRIT-S surveys.

### Mental training intervention

The mental training intervention was designed to examine the primary research question regarding the physiological underpinnings of traditional mental skills training. Mental training can also be called psychological skills training, and, as defined by the American Psychological Association, is a program of instruction and practice in the use of relaxation, concentration, imagery, goal setting, and energizing to enhance athletic training. A Certified Mental Performance Consultant © (CMPC) who has more than 10 years of field experience working with endurance and team athletes designed the mental training protocol. The mental training protocol was video recorded to ensure consistent delivery to participants.

The mental training intervention consisted of four videos containing exercises for participants to use to enhance their performance: an introduction to mental skills training and breathing techniques to reduce stress and anxiety while allowing increased confidence and feelings of well-being (Wilson and Taylor 2014) (video time 9 min and 43 s), a “controlling the controllables” lesson to focus more on controllable aspects of performance to reduce stress (video time 8 min), self-talk and confidence intervention to combat negative thinking and doubt (video time 11 min and 25 s), and finally, an imagery intervention to mentally prepare for performances and challenging moments (video time 4 min and 40 s). The goal of the mental training intervention was to equip participants with breathing techniques, cognitive behavioral strategies, and mental preparation strategies to endure fatigue and enhance endurance. Each of the videos, other than the introduction, were watched two times during the week at home for a total of 3 weeks. The introductory video was watched three times in total, immediately after each TTE on days 3–5. In addition, the participants were instructed to follow along with the video, performing the instructed activity, to practice the different skills being taught and to keep them as active listeners. Adherence to watching the videos was assessed weekly by asking participants a question on what they had found most interesting/beneficial from the videos for that week. Total time of the mental training intervention was 173 min and 39 s. The control group were not given any videos during the study. However, each CON participant was given the videos at the end of the study.

### Psychological measures

The ten-item Connor–Davidson Resilience Scale (CD-RISC; (Connor and Davidson 2003)) was employed to measure resilient characteristics in the participants. The 10-item CD-RISC was used in place of the original 25-item scale because the 10-item scale has stronger psychometric properties in sport and performance contexts (Gucciardi et al. 2009; Gonzalez et al. 2016)). Participants were directed to indicate how much they agreed with statements as they apply to their lives. Each response was given on a five-point Likert-Type Scale (0—*not at all true* to 4—*true nearly all the time*). Example items included “I can deal with whatever comes my way,” Having to cope with stress can make me stronger,” and “I tend to bounce back after illness, injury, or other hardships.” Scores were summed and ranged from 0 to 40 with higher totals indicating more resilient characteristics. Cronbach’s alpha for the CD-RISC in this study was 0.778 at pre-test and 0.862 at post-test.

Grit was assessed with the Short Grit Scale [Grit-S; (Duckworth and Quinn 2009)]. The Grit-S consists of two, four-item subscales that measure interest and effort. Each participant was asked to answer the following statements honestly on a five-point Likert-Type scale (1—*not at all like me* to 5—*very much like me*). Examples of items included “I have difficulty maintaining my focus on projects that take more than a few months to take” (interest subscale) and “Setbacks don’t discourage me” (effort subscale). Scores were summed for each subscale and ranged from 4 to 20 with higher totals indicating more interest or effort. Cronbach’s alphas for the interest subscale was 0.765 at pre-test and 0.616 at post-test and for the effort subscale was 0.853 at pre-test and 0.507 at post-test.

### Neuromuscular testing

Central and peripheral contributions to muscle fatigue, 5–10 min before and 1 min after each exercise test (TTE), were examined by superimposing a supramaximal magnetic stimulation of the femoral nerve during and 5 s after a series of maximal voluntary contractions (MVCs) of the quadriceps. Participants sat in a semi-reclined position on a table, with the upper body and lower back supported at a hip angle of 45°, and the knee joint angle set at 90° of flexion with the arms folded across the chest. A magnetic stimulator (Magstim 200^2^; Wales, UK) connected to a 70 mm double coil was used to stimulate the femoral nerve. The evoked quadriceps twitch force was obtained from a calibrated load cell (MLP-300; Transducer Techniques, Rio Nedo Temecula, CA) connected to a noncompliant strap which was placed around the participant’s right leg, just superior to the malleoli.

Maximal femoral nerve stimulation was verified in each participant by assessing unpotentiated quadriceps single twitch forces obtained at 70%, 80%, 85%, 90%, 95%, and 100% of maximal stimulator output. A plateau in baseline unpotentiated quadriceps single twitch force with increasing stimulus intensities was observed in every participant and a plateau in M-wave amplitudes was observed in the sub-set of participants in which EMG was recorded. The stimulator was set at 100% for all participants and trials.

A superimposed twitch force during and a potentiated force (*Q*_tw,pot_) 5 s after a 5-s maximal isometric voluntary contraction of the quadriceps were measured and this procedure was performed six times. Like others, we found the degree of potentiation was slightly smaller after the first and second MVC (Kufel et al. 2002); therefore, we discarded the first two measurements. Peak force, maximal rate of force development (MRFD), contraction time (CT), and reaction time (RT_0.5_) were analyzed for all Q_tw,pot_. Voluntary activation of the quadriceps during the MVCs was calculated using the following equation: 1 − (superimposed twitch force/*Q*_tw.pot force_)*100.

### Electromyography

Quadriceps electromyogram (EMG) was recorded from the right rectus femoris (RF) using monitoring electrodes with full-surface solid adhesive hydrogel (Delsys Trigno Wireless EMG, Natick, MA, USA) with on-site amplification. Electrodes were placed in a bipolar electrode configuration on the midpoint of the RF with an inter-electrode distance of 100 mm. The EMG electrode was placed in the same location during all visits. The surface EMG electrodes were used to assess the maximal EMG of the RF during a maximal voluntary contraction before each TTE. RF EMG was continuously measured during the subsequent time trial to estimate changes in central neural command.

All EMG recordings were high-pass filtered using fourth order zero-lag Butterworth filters and subsequently smoothed using a root-mean-square (RMS) filter (30-ms symmetrical moving window with successive 1-ms steps). EMG signal amplitudes from the TTE were normalized to the maximum RMS EMG amplitude recorded during MVC testing of the quadriceps. The EMG amplitudes during the TTE were measured over five seconds at baseline and then measured as an average of 0–20%, 20–40%, 40–60%, 60–80%, and 80–100% based on the shorter trial. Due to recording issues (i.e. electrode falling off/coming loose, etc.), data from six participants were excluded from analysis.

### Perceptual measures

Perceptual responses for leg specific fatigue and leg specific pain during each TTE were recorded every minute. Numerical ratings of leg fatigue and pain, from 0 to 100 were used to assess the severity of preexisting and exercise-related leg fatigue and pain symptoms, comparable to “perceived discomfort” scales described by (Christian et al. 2014). Fatigue and pain ratings were anchored with 0 being described as no pain or fatigue, 25 being described as mild pain or fatigue, 50 being described as moderate pain or fatigue, 75 as severe pain or fatigue, and 100 as the worst possible fatigue or pain imaginable. Participants provided ratings of perceived exertion (RPE) every 30 s using the Borg 6–20 scale (Borg 1982), explained as the answer to the question “how hard do you feel like you are working?” thus representing the combination of effort and peripheral sensations.

### Statistical analysis

Data for all physiological and perceptual measurements were recorded using the “individual isotime” method as described by Nicolò et al. (2019) in order to compare each test at the same absolute timepoint. In short, the worst of the two TTE tests was used to identify 11 timepoints into which the two tests were segmented and normalized as a percentage of the shortest TTE (0–100%). For all participants, each time point varies from the other participants on the basis of their worst TTE.

Time to exhaustion and neuromuscular data were analyzed using 2 (treatment) × 2 (trial) repeated measures ANOVAs using SPSS version 28. Significant treatment effects and time by treatment interactions were followed up with post hoc paired *t* tests.

Physiological data were analyzed using 2 (treatment) × 2 (trial) × 11 (time) repeated measures ANOVA. To evaluate treatment and time effects for EMG data, a 3 (treatment) × 2 (trial) × 6 (time) repeated measures ANOVA was performed. In instances where Mauchly’s test for sphericity was significant, Hyunh-Feldt correction was used to adjust for degrees of freedom. If time effects were significant, planned contrasts were used to determine which points differed from baseline. Significant treatment effects and treatment by time interactions were followed up with post hoc paired *t* tests. Psychological data were analyzed using a repeated measures ANOVA. All data were presented as means and standard deviations, with significance set at α < 0.05.

## Results

### Group characteristics

Age, VO_2peak_, PPO (peak power output), TTE workload, and TTE workload as a percentage of PPO were not statistically different (*p* > 0.05) between groups (see Table 1).

**Table 1:** Baseline data for MT and CON

	Age (years)	VO_2peak_ (ml/kg/min)	PPO (W)	TTE workload (W)	TTE (% of PPO)
MT (*n* = 16)	20.75 ± 1.0	47.9 ± 9.0	255.3 ± 50.0	223.0 ± 43.5	87.5 ± 4.6
CON (*n* = 17)	20.71 ± 1.5	47.9 ± 9.9	263.2 ± 69.4	230.1 ± 64.9	87.2 ± 6.2

### Effects of mental training on time to exhaustion

**Fig. 1:**
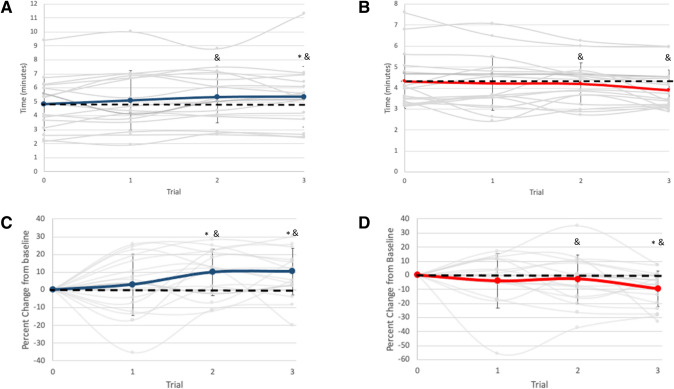
Mean pre- to post-training changes in time to exhaustion times for **A** MT (blue) and **B** CON (red) groups and individual values (gray lines). Percent change for the TTE tests are shown for **C** MT and **D** CON groups. * indicates a significant difference between groups (*p* < 0.05) and & indicates a significant change from baseline (*p* < 0.05)

As predicted, mental training had a significant effect on time to exhaustion (Group × Time interaction, *F* (3,96) = 4.14, *p* = 0.008), as shown in Fig. 1. A post hoc test revealed that time to exhaustion was only improved as compared to control following 2 weeks of mental training, increasing from a pre-training time of 4.85 ± 1.92 min to a time of 5.33 ± 1.83 min after 2 weeks (*p* < 0.05) and 5.35 ± 2.17 min after 3 weeks of mental training (*p* < 0.05). The CON group remained unchanged from a baseline time of 4.32 ± 1.31 min to 4.20 ± 1.00 min after 2 weeks of mental training (*p* > 0.05) before significantly decreasing to 3.90 ± 0.96 min during the final TTE (*p* < 0.05).

When TTE was controlled for the initial length of the time trial by examining change as a percentage of initial TTE, the significant improvement in TTE from MT remained, [Group × Time, *F* (3,93 = 4.35, *p* = 0.006) (10.03 ± 13.1% improvement after 2 weeks; 10.44 ± 13.2% improvement after 3 weeks (*p* < 0.05)] as did the significant decrement in performance after 3 weeks of CON [−10.3 ± 12.7% after 3 weeks (*p* < 0.05); −3.2 ± 17.3% after 2 weeks (*p* > 0.05)], as shown in Fig. 1.

### Effect of mental training on physiological variables

#### Metabolic variables

**Fig. 2:**
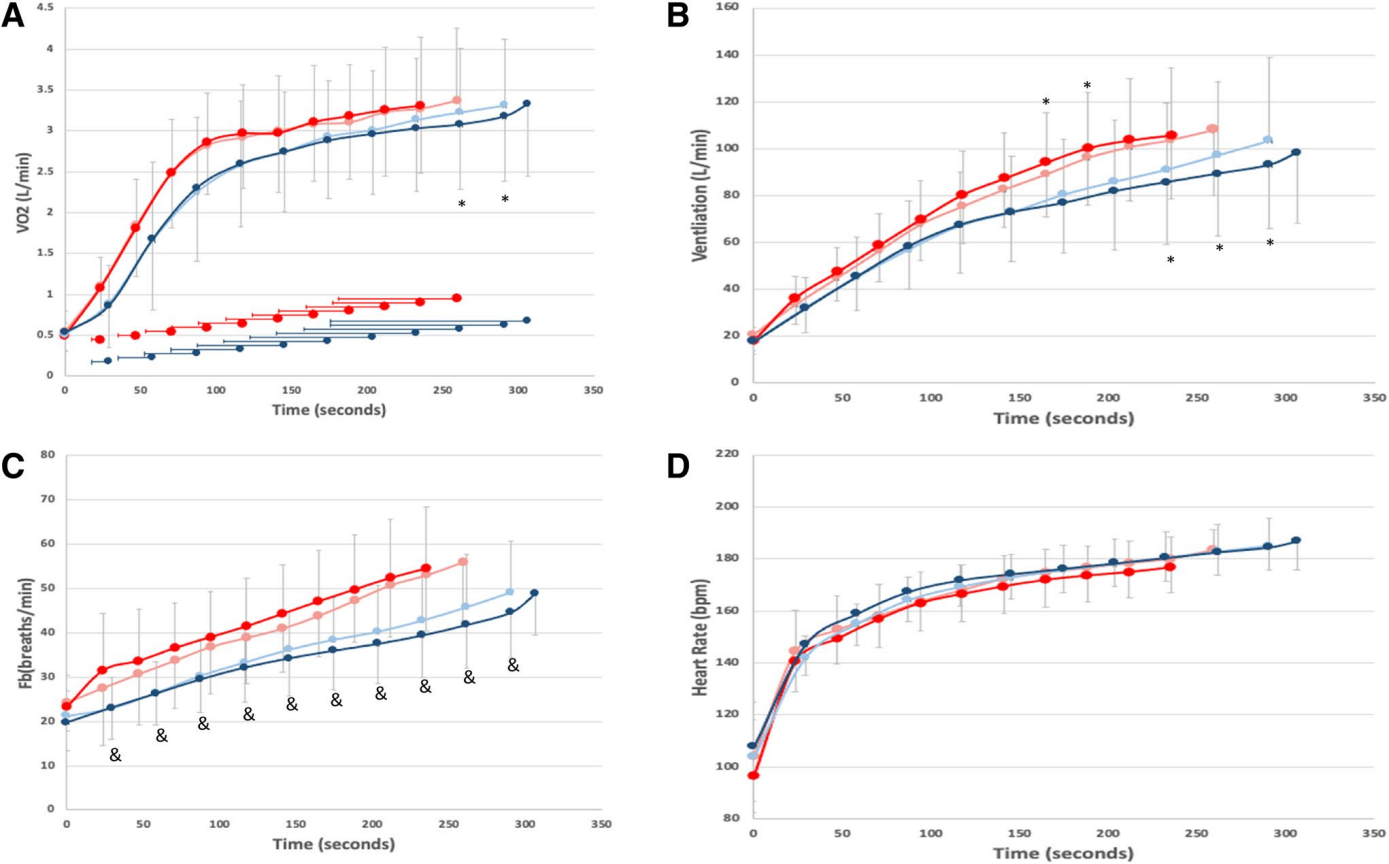
Mean ± SD for **A** VO_2_, **B** ventilation, **C** respiratory rate (*F*_b_), and **D** heart rate for pre- (light line) and post-training (dark line) for MT (blue) and CON (red). * indicates a significant difference between trials (*p* < 0.05) and & indicates a significant group response during trial the post-TTE (*p* < 0.05)

Initial analysis of VO_2_ data revealed a significant Trial × Time × Group interaction (*F*(6.18,310) = 2.253, *p* = 0.038). Post hoc analysis revealed that VO_2_ was significantly lower following MT training during the last two measured time points of the TTE (90% and 100% of shortest time trial, *p* < 0.05, Fig. 2). VO_2_ decreased from 3.22 ± 0.78 to 3.07 ± 0.78 ml/kg/min at 90% and from 3.31 ± 0.82 to 3.17 ± 0.79 ml/kg/min at 100% of the shortest TTE. The maximum VO_2_ for the longest TTE was the same as the maximum VO_2_ for the shortest TTE. For CON, there was no difference in VO_2_ between the two trials.

Ventilation also displayed a significant Trial × Time × Group interaction (*F*(3.75, 310) = 4.85, *p* = 0.002). Post hoc analysis revealed that ventilation was significantly reduced following MT during the last three measured time points (80–100% of the shortest time trial, *p* < 0.05). MT training reduced ventilation by 5.5 ± 10.1, 8.1 ± 10.9, and 10.5 ± 12.4 L/min at 80%, 90%, and 100% respectively. After 3 weeks of CON, ventilation was significantly increased at 60% and 70% of the shortest TTE (*p* < 0.05), 4.7 ± 8.5 and 5.1 ± 9.1 L/min respectively during the last TTE (Fig. 2).

For respiratory rate (*F*_b_), initial analyses revealed a Trial × Group interaction (*F*(1.0, 31) = 7.43, *p* = 0.01). Post hoc analysis revealed that respiratory rate was significantly increased in CON, while it was reduced following MT. For tidal volume, there were no differences detected for either MT or CON between pre-training and post-training.

There were no differences found between trials for heart rate. The Trial × group interaction approached significance (*F*(1, 31) = 3.178, *p* = 0.084) with heart rate trending toward slight increases in the CON group from pre- to post-training (Fig. 2).

#### Electromyography

**Fig. 3:**
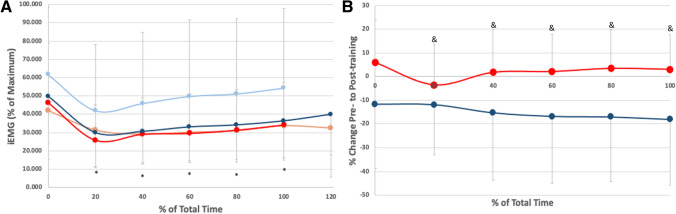
Mean ± SD for **A** iEMG as a percent of maximum and **B** percent change in EMG from pre-(light line) and post-training (dark line) for MT (blue) and CON (red). * indicates a significant difference from pre- to post-training for MT and & indicates a difference between groups

Figure 3 depicts how EMG changes with training. There were no differences at baseline between the CON and MT groups (*F*(1, 24) = 0.261, *p* = 0.614). Analysis revealed a significant Trial × Group interaction (*F*(1, 23) = 5.294, *p* < 0.05) with post hoc analysis revealing that there was a significant decrease in EMG due to mental training at all time points other than the initial time point (time point 0). The mean difference between MT and CON was −18.19%, which represented a significant decrease in the electrical activity of the rectus femoris (*p *< 0.05).

#### Contractile properties of the muscle

Immediately after each time trial, group mean *Q*_tw.pot_ was reduced from pre-exercise baseline (*p* < 0.01). Exercise-induced changes in quadriceps muscle function were similar following both MT and CON (Table 2). However, the rate of fatigue accumulation, calculated as the decrement in *Q*_tw.pot_ divided by the time to exhaustion showed a significant group difference between the pre- and post-training TTE (*F*(1, 31) = 6.054, *p* < 0.05) as fatigue accumulated less quickly following MT, but more quickly within CON. MVC force measurements were also altered from baseline immediately after each time trial (*p* < 0.05), and there were no changes following either 3 weeks of MT or CON. There were no differences in voluntary muscle activation (VMA) for MT (69.4 ± 19.2% vs 69.5 ± 13.6%) but CON had a significant decrease following the pre-training TTE (69.1 ± 18.6% vs 61.6 ± 18.1%, *p* < 0.05) after exercise. Similarly, after 3 weeks of mental training, there were no differences in VMA for MT following exercise (73.4 ± 16.8% vs 69.2 ± 19.3); however the significant decrease in VMA remained in the CON (72.7 ± 17.0 vs 65.5 ± 16.8%, *p* < 0.05). There were no significant differences between MT and CON in any of the within-twitch measurements (MRFD, MRR, CT, and RT_0.5_).

**Table 2:** Peripheral fatigue was assessed via supramaximal magnetic stimulation of the femoral nerve before and 1 min after exercise

	Percentage change from pre- to post-TTE			
	CON		MT	
	Pre-	Post-	Pre-	Post-
*Q*_tw.pot_ (N)	− 46.9 ± 24.9	− 47.2 ± 14.7	− 37.8 ± 16.1	− 41.7 ± 19.7
MRFD (N s^−1^)	− 48.2 ± 34.4^$^	− 48.3 ± 22.2^$^	− 35.8 ± 25.5^$^	− 39.8 ± 33.1^$^
MRR (N s^−1^)	− 35.2 ± 32.9^$^	− 43.5 ± 22.5^$^	− 36.5 ± 27.0^$^	− 30.9 ± 24.1
CT (s)	− 2.6 ± 13.1^$^	− 6.3 ± 14.0^$^	− 7.3 ± 20.5^$^	− 2.6 ± 18.9^$^
RT_0.5_ (s)	− 12.5 ± 26.4^$^	− 9.9 ± 19.1^$^	− 5.9 ± 20.5^$^	− 2.5 ± 19.5^$^
MVC peak force (N)	− 11.0 ± 22.5	− 14.9 ± 15.2	− 13.9 ± 14.7	− 16.0 ± 11.6
Percentage voluntary muscle activation	− 3.6 ± 16.7^$^	− 8.8 ± 10.5	− 9.4 ± 18.9	− 5.5 ± 17.0^$^
Rate of fatigue accumulation (*Q*_tw.pot_/TTE)^&^	− 10.9 ± 5.8	− 12.5 ± 4.3	− 8.4 ± 4.4	− 8.1 ± 3.6

Changes in fatigue variables are expressed as a percentage change from pre-exercise baseline to 1 min after the completion of TTE. Values are expressed as mean ± SD

*Qtw.pot* potentiated single twitch; *MRFD* maximal rate of force development; *CT* contraction time; *RT0.5* one-half relaxation time; *MVC* maximal voluntary contraction

^$^ indicates no significant decrease from the pre-exercise baseline (*p* < 0.05). Indicates no difference from baseline

^&^ indicates a significant between-subjects effect (*p* < 0.05)

### Effect of mental training on perceptual variables

**Fig. 4:**
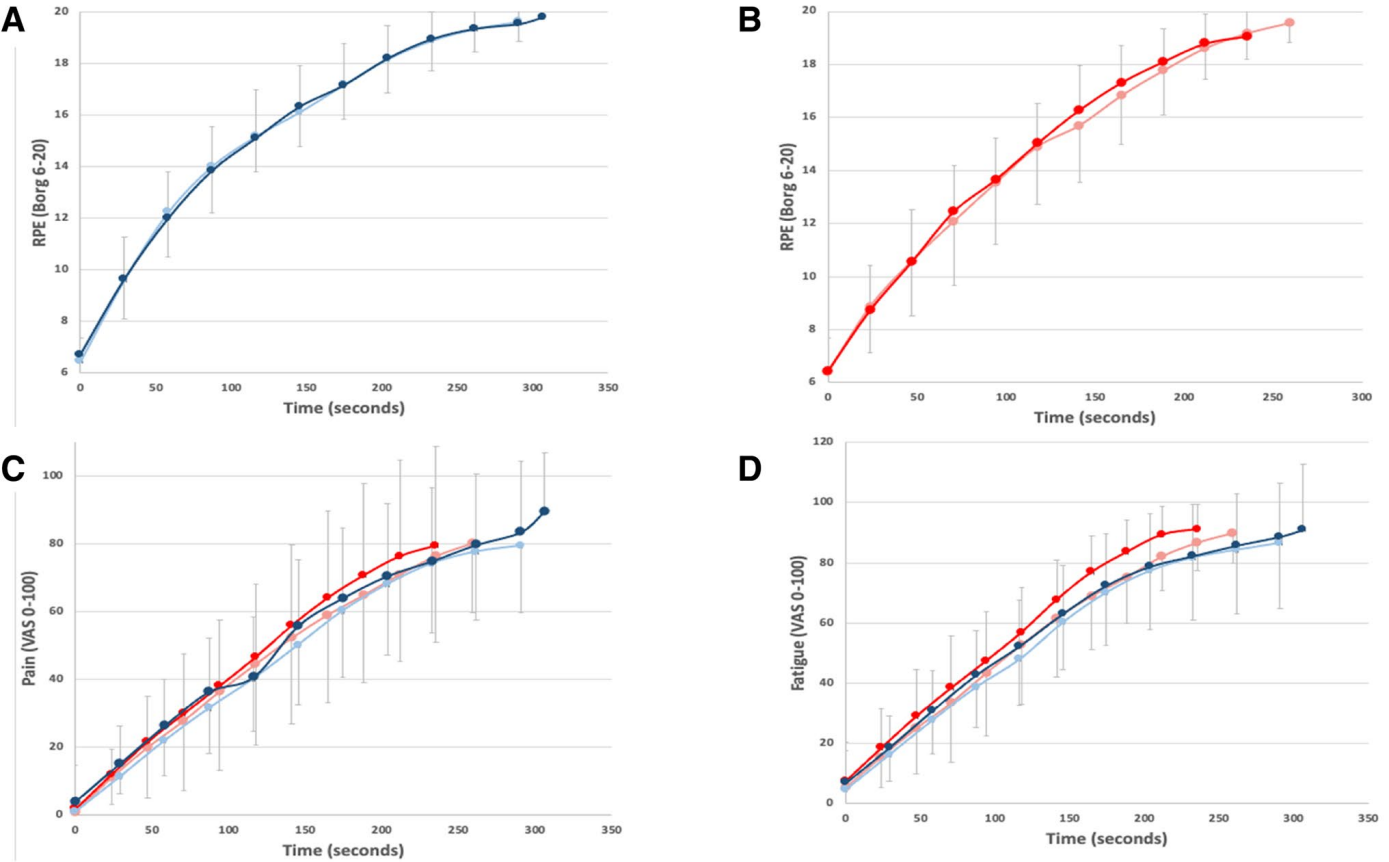
Mean ± SD for **A** RPE in the MT group, **B** RPE in the CON group, **C** perception of leg pain (VAS 0–100 scale), **D** perception of leg fatigue (VAS 0–100 scale) for pre-training (light line) and post-training (dark line) for MT (blue) and CON (red)

Mental training did not affect rating of perceived exertion as analysis revealed that there was no Trial × Time × Group interaction (F(10,290) = 0.548, *p* = 0.855). This suggests that mental training did not reduce the perceived exertion of exercise at any given time point (Fig. 4). However, the CON group did have a significantly lower maximal RPE during the post-trial (19.1 ± 1.1) as compared to the pre-trial (19.6 ± 0.73, *p* < 0.05).

Similar to the rating of perceived exertion, neither the sensation of leg pain (*F*(10,290) = 0.803, *p* = 0.509) nor the sensation of leg fatigue (*F*(10,290) = 0.795, *p* = 0.486) was reduced by mental training (Fig. 2). There were also no differences between the maximal levels of leg pain or fatigue observed between either of the trials in either of the groups (*p* > 0.05).

### Effect of mental training on psychological variables

Between pre- and post-time trials, grit as an overall construct was unchanged for both MT and CON (*F*(1,28) = 0.01, *p* > 0.05). And, when the individual constructs of grit were analyzed, neither perseverance of effort nor consistency of effort differed between trials (Table 3). For resilience, there was no trial or trial × treatment interaction, though MT approached reaching a significant increase as resilience improved from a pre-training value of 28.9 ± 4.80 to a post-training value of 30.4 ± 4.13 (*p* = 0.072).

**Table 3:** Total GRIT-S questionnaire scores as well as the sub-categories for grit which include consistency of interest and perseverance of effort

	MT		CON	
	Pre-	Post-	Pre-	Post-
Grit				
Consistency of interest	13.3 ± 3.24	12.9 ± 3.38	12.6 ± 3.13	12.4 ± 3.72
Perseverance of effort	15.4 ± 2.13	15.7 ± 2.24	15.7 ± 1.98	15.6 ± 2.82
Total	28.7 ± 4.73	28.6 ± 5.15	28.3 ± 4.66	28.1 ± 6.24
Resilience				
Total	28.9 ± 4.80	30.4 ± 4.13	31.1 ± 4.19	31.3 ± 6.29

## Discussion

This study investigated the effects of mental training on time trial to exhaustion performance and attempted to elucidate the physiological or perceptual mechanisms by which MT may alter performance. As hypothesized, time trial performance was increased, though 2 weeks were needed for this performance increase to become apparent, differing from a previous study in our lab where an 8.5% increase in time trial to exhaustion occurred after only 1 week of mental training (Van Haitsma et al. 2019). No further benefit from mental training occurred with an additional week of practice during this study. Physiologically, significant decreases with MT occurred in electromyography and several metabolic variables, though no changes were seen in perceptual variables.

Like our previous study that found an 8.5% improvement in time trial to exhaustion performance, this study found a 30 s, or 10.4%, improvement in TTE with MT training. These findings are supported by other endurance-based psychological interventions. Motivational self-talk, used on its own, yielded an 18% improvement in performance though the relative intensity was 7% lower and the TTE was more than twice as long (Blanchfield et al. 2014). Similarly, in a study that did mental training utilizing 4 1-h sessions before the final trial, participants ran 8% further with mental training (Barwood et al. 2007).

### Effect of mental training on physiological outcomes

The goal of the MT program was to equip participants with strategies to endure fatigue and enhance endurance by modifying the mental, or central, approach to exercise. These strategies included breathing techniques to both activate the parasympathetic system and/or reduce the activation of the sympathetic system as well as thinking strategies for preparation for and execution of time trial to exhaustion (Jones 2002; Birrer and Morgan 2010). By training arousal regulation and reducing activation of the sympathetic system prior to exercise, participants may have been able to decrease pre-exercise anxiety (Evans et al. 2004). In addition, by utilizing imagery within training, MT participants may have been able to increase motivation, particularly in week 3 when motivation and performance appeared to be reduced in CON participants (Paivio 1985).

By using these techniques, one of the primary changes that occurred was a 10–20% reduction in rectus femoris EMG during the post-mental training time to exhaustion trial. Mental training has been suggested to improve motor flexibility, perhaps improving the ability for the cerebellum to select the proper motor behavior (Olsson et al. 2008), allowing for a better kinematic, or neuromuscularly efficient, cycling pattern (Lohse et al. 2010; Gentili et al. 2010). This may decrease the number of errant electromyography signals that reach the rectus femoris, reducing EMG signal. Imagery training may also shift the locus of control from an internal, muscle focus to an external movement effect focus, reducing EMG activity (Vance et al. 2004; Zachry et al. 2005). By reducing EMG activity, muscle synchronization may be decreased (Yao et al. 2000), smoothing the power output of skeletal muscle, allowing for glycogen sparing (Osborne and Schneider 2006) and subsequently reducing the rate of fatigue accumulation though final peripheral fatigue is unchanged.

A reduction in muscle activation as measured by EMG may also have effects on other physiological variables as EMG is traditionally used as a marker of central motor command (Carrier et al. 2011; Gaveau et al. 2021). While there was no difference in metabolic parameters for the initial, or primary, periods of exercise, there was a decrease in VO_2_ and ventilation during the latter periods of exercise that are consistent with changes to the VO_2_ slow component. It would not be expected to see a difference in the primary VO_2_ response to exercise as the primary phase is largely dependent on the external power requirement which was consistent between trials (Paterson and Whipp 1991). However, the VO_2_ slow component is thought to reflect a decrease in the efficiency of muscle contractions, perhaps due to the recruitment of more Type II muscle fibers, leading to an increase in metabolic demand (Jones et al. 2011). Mental training appears to modify the central demand, reducing EMG, and improving muscle efficiency as evidenced by the reduced VO_2_ slow component in the MT participants.

Arousal regulation training may have also led directly to some physiological changes within the post-training TTE. In one of the videos watched by the participants, they were instructed to “breathe deep with their belly as well as slow down the cadence of breathing”. By following these instructions, participants were able to reduce their ventilation which increases parasympathetic activity and decreases sympathetic activity (Cottin et al. 1999; Pal and Velkumary 2004). By increasing parasympathetic activity, participants may have better decision control during the trial in order to keep cycling by increasing inhibitory control from the prefrontal cortex (Forte et al. 2021), leading to decreased amygdala activation and decreased emotional reactivity, even amid the discomfort of a cycling trial (Bechara et al. 2005). In addition, by reducing the activation of the amygdala-parabrachial circuit (Luskin et al. 2021), which can function as a respiratory pacemaker (Felten et al. 2021), and by reducing anxiety, frequency of breathing has been shown to be decreased (Masaoka and Homma 2001). Conversely, when anxiety is increased during exercise through negative self-talk, ventilation and frequency of breathing was increased through a similar pathway (Basset et al. 2021).

### Effect of mental training on perceptual outcomes

Rating of perceived exertion was not changed with mental training despite a decrease in rectus femoris EMG. During both pre- and post-mental training, RPE increased at the same rate and ended at the same absolute value. A previous self-talk training intervention similarly found that there was not a decrease in RPE after training (Basset et al. 2021) while a different study found that positive self-talk reduced RPE (Blanchfield et al. 2014), but EMG was not measured during either trial. While perception of effort may reflect central motor command during a unilateral elbow flexion exercise (de Morree et al. 2012), during an endurance trial to exhaustion, there may be other factors that affect RPE. For example, vibration has been demonstrated to reduce RPE without changing central activation during exercise (Shibuya et al. 2009).

Likewise, perceptions of leg specific fatigue and pain during the time trial were not changed by mental training nor were end-exercise maximal levels of fatigue and pain. As the time trial to exhaustion consists of an exercise at a fixed intensity until failure, it would be expected that there would be no difference with mental training in leg fatigue and pain. During exercise, it is thought that leg fatigue and pain are caused by activation of the Group III/IV afferent neurons by the combination of several metabolites including lactate, ATP, and protons (Light et al. 2008; Pollak et al. 2014); and as long as fitness and peripheral load remains unchanged, these metabolites should increase at the same rate in each trial. The fact that RPE and perception of leg fatigue and pain were not changed, while EMG was reduced, suggests that RPE may be influenced heavily by peripheral sensations, not just central feed-forward mechanisms (Marcora 2009).

### Mental training and psychological outcomes

Three weeks of mental training had no effect on grit or either of the two subscales, which reflects our previous 1-week mental training intervention (Van Haitsma et al. 2019). The grit scale was originally designed to work in a general domain, examining both the consistency of interest and the perseverance of effort in the pursuit of a long-term goal (Duckworth et al. 2007). However, a time trial to exhaustion is a short-term goal, and grit has been previously shown to be a stable trait characteristic as demonstrated in a group of individuals who took the GRIT-S twice, separated by one year (Duckworth and Quinn 2009). This suggests that the self-report measures are unable to detect the subtle mindset changes that occur following mental training and that may affect athletic performance. There is also the possibility that 3 weeks of mental training are not of long enough duration to cause a psychological change that is measurable by the current psychological scales, though 12-weeks of imagery training has been shown to change grit (Rhodes et al. 2018).

While resilience did not significantly increase from 3 weeks of mental training, it did approach a significant increase. Resilience can be defined as “the role of mental processes and behavior in protecting an individual from the potential negative effect of stressors” (Galli and Gonzalez 2015). In this study, all individuals fail at some point, which is marked as the end of the trial. However, they were never given feedback about the duration of their TTE, whether they performed better or worse than during their original performance, meaning that the participant never knew if they were facing a negative stressor (Gonzalez et al. 2018). Participants just knew that the adversity was the performance of the TTE, which could be taken as either a positive or negative stressor.

One interesting finding during this study was the significant 10% decrease in performance in the control group during the third week of TTEs. Of the 17 individual participants in the CON group, only four individuals had an increase in performance while some individuals had as large as a 30% decrease in TTE performance. By not receiving any mental training, the CON group may have had a decrease in their desire to persevere, even though the psychological values were consistent over time. While the coefficient of variation for a time trial to exhaustion is fairly high (Jeukendrup et al. 1996), the decrease in performance exceeds the typical coefficient of variation and suggests that psychological factors such as disinterest or boredom in the trial may play a role in the performance decrement.

### Limitations

There were several limitations to this study. First, a time trial to exhaustion was used rather than the more repeatable fixed distance time trial as the marker for performance (Jeukendrup et al. 1996). While a fixed distance time trial would allow for pacing, pacing would increase variance within the physiological variables, masking any changes that may occur due to the psychological training. Second, this study performed electromyography on the rectus femoris, rather than the vastus lateralis, as a proxy for the whole leg during cycling. While the vastus lateralis is typically used during cycling if only one electrode is used, the rectus femoris mirrors that of the vastus lateralis during constant-load intense cycling (Decorte et al. 2012).

## Conclusions

In summary, 3 weeks of mental training modifies central motor command as evidenced by a decrease in rectus femoris EMG. This reduction produced lower ventilation, oxygen consumption, and rate of fatigue accumulation as measured by potentiated twitch. These physiological changes led to a 10% increase in performance in a cycling time to exhaustion test and prevented a decrease in performance as seen in the control group. Further study is necessary to determine what exact changes are occurring within the brain and to verify the physiological and psychological improvements following mental training.

## Data Availability

The datasets generated and analysed during the current study are available from the corresponding author on reasonable request.

## Abbreviations

MT: Mental training
CON: Control
TTE: Time trial to exhaustion
VT: Ventilatory threshold
EMG: Electromyography
VO_2_: Volume of oxygen consumption
VE: Ventilation
MVC: Maximal voluntary contraction
Q_tw.pot_: Potentiated twitch force
RPE: Rating of perceived exertion
CT: Contraction time
RT_0.5_: Half relaxation time
RF: Rectus femoris
PPO: Peak power output
*F*_b_: Respiratory rate
